# Colonels and generals, chairs and deans: How the military educates its leaders and what we can learn in academic medicine

**DOI:** 10.3389/frhs.2023.1075543

**Published:** 2023-02-20

**Authors:** Peter Nagele

**Affiliations:** Department of Anesthesia and Critical Care, University of Chicago, Chicago, IL, United States

**Keywords:** leadership, management - healthcare, education, military, professional

## Introduction

While the professions may be profoundly different, successful physician leaders and military officers will rise to lead organizational units with increasing complexity and size over the course of their careers. Military officers are required to participate in professional military education and its successful completion is a mandatory requirement for promotion. Professional military education spans the whole career, is robust, extremely well designed and continually reviewed and updated. The goal of this article is to inform how the US military has successfully structured professional military education and to discuss how we may apply a similar approach to the leadership development and education of academic physician leaders. The focus of this article is on physician leadership in academic medical centers due to the complexity of managing a healthcare business and an academic enterprise, but the concepts are broadly applicable to all leaders in healthcare in academic and non-academic settings.

Using the US Army as an example, I will briefly review the organizational structure of modern armies, review requisite skill sets and expectations for US Army officers at different echelons (organizational levels), and review how the US Army has designed professional military education and training for officers over the course of their careers. The basic concepts are very similar in other branches of the US military (e.g., Navy, Air Force) and armed forces of other Western nations.

## Military echelons—from platoon to field army

For simplicity, we will use infantry as example to illustrate the military organizational hierarchy (“levels of command” or echelons) (1) and use a typical career path of an US Army infantry officer, starting at the junior officer level (Table 1).

**Table 1:** Military levels of command/echelons (in increasing size and complexity) with typical leader rank—using infantry as an example.

Military element	Number of soldiers (range)	Leader rank
Squad	7–10	Sergeant
Platoon	35–45	Lieutenant
Company	150–250	Captain
Battalion	800–1,200	Major/ Lieutenant Colonel
Brigade	3,500–5,000	Colonel/Brigadier General
Division	15,000–20,000	Major General
Corps	50,000+	Lieutenant General
(Field) Army	150,000+	General

Infantry is the original form of military force and conducts ground combat. The smallest infantry unit is a squad, which is typically lead by a non-commissioned officer (typically a sergeant). The first infantry unit a new officer graduate (second lieutenant) will command is an infantry platoon. An infantry platoon consists of approximately 40 soldiers (typically split into 3–4 squads) with little if any formal command and control or logistical support elements. The requisite skill set is small unit tactics, and the expectation is that the platoon be ready and capable of completing any mission assigned. Leadership at the platoon level is immediate, personal, and direct with a focus on “doing” and achieving mastery of the unit's weapon systems and performance in combat. The next echelon is an infantry company (150–200 soldiers), commanded by a captain, and typically comprised of 3 infantry platoons and one heavy weapons platoon. For an army officer, this is the first time they have a formal headquarters unit and a logistical support element. The requisite skills include being able to direct and lead platoons, use the headquarters as an asset to generate the best plans and provide the highest level of support (supply, intelligence) to the platoons. Leadership at the company level remains immediate and personal (Table 2).

**Table 2:** Comparison of military and AMC leadership.

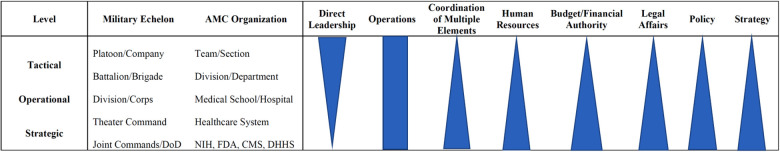

Leading at the next level of combat, the infantry battalion (500–1,000 soldiers), is much more complex. A battalion, comprised of 3–5 companies and commanded by a lieutenant colonel, is the smallest army unit that has a formal headquarters staff organization with staff officers responsible for human resources (S-1), intelligence (S-2), operations (S-3), logistics (S-4), communications (S-6), a supply/logistics (headquarters) company and several other support elements for which the battalion commander no longer is a formally trained expert. The headquarters staff also includes an executive officer (typically a major) and a senior non-commissioned officer (NCO). The coordination of support elements, such as mortar/artillery, scouts, snipers, air-defense, combat engineers, a medical platoon, with the main fighting force in combat is complex and requires a high level of expertise and training. At the battalion level, leadership transitions from direct to more indirect leadership *via* staff officers and company commanders.

An infantry brigade, the next military echelon, has 3,000–5,000 soldiers and consists of 3–5 infantry battalions and has even more disparate support elements than an infantry battalion. An infantry brigade, typically commanded by a colonel (sometimes by a brigadier/1-star general) is the central combat maneuver unit of the US Army. The slightly larger infantry brigade combat team (IBCT) is composed of 7 battalions, including three infantry, cavalry, artillery, engineering, and support battalions. The ability to plan and support combat operations at the battalion level are requisite skills for a brigade commander, who must also understand how to allocate artillery and engineering assets to best support the brigade's mission. The brigade commander is also tasked with coordinating operations with forces from other branches of the army (e.g., armored & mechanized infantry units) and aviation support (from the army or the air force). A brigade commander straddles the boundary between tactical and operational levels of warfare (Table 2).

An infantry division (10,000–20,000 soldiers), commanded by a two-star general, consists of three infantry brigades and similarly a significant amount of command, control, communications, planning, intelligence, and other support elements, and is an operational command. Divisions conduct large-scale operations that can span 50–100 miles and involve highly complex combined warfare operations (Table 2).

The final organizational unit that the United States Army deploys as a maneuver unit is the field corps (commanded by a 3-star general). A field corps (size: >50,000 soldiers) executes theater-level operations, and its leadership requires a deep understanding of joint military operations involving multiple military services (e.g., Army, Navy, Air Force), military strategy and policy, and interfaces closely with central military planners in US combatant commands and the Department of Defense (Table 2). The U.S. military has higher echelons in its formal organization, such as field army, unified combatant commands, the Joint Chief of Staffs, and ultimately the Department of Defense. These organizational units do not deploy as maneuver units in a theater of war and provide command and control and administrative coordination.

Thus, over the span of a 25-year career, an infantry officer may lead units as small as 40 soldiers and as large as several tens of thousands of troops. How does the military train and educate its leaders to be effective and successful at each echelon when the demands and qualifications are so different? The answer is that the US military invests substantial resources towards professional military education with multiple rounds of mandatory formal education interspersed with what could be referred to as training on the job (2). The next section will take a detailed look at professional military education for U.S. infantry officers.

## Professional military education

A typical career as active-duty infantry officer begins after graduating from college and being commissioned as second lieutenant in the Army. Successful active-duty Army officers stay in the military for 20 years at which point they will have attained the rank of lieutenant colonel and be eligible for retirement (3). A select few officers are selected to continue their active-duty career and are promoted to the rank of colonel (and subsequently to a general officer rank), which allows them to remain in the army until the mandatory retirement age at 62 (a 40-year career) and continue their career as general officers (brigadier general (one-star), major general (two-star), lieutenant general (three-star), and general (four-star). Four-star general is the highest rank in the peacetime United States military.

After graduating from United States Military Academy, active-duty US Army officers receive more than 30 months of mandatory formal military leadership education during their typical 25-year career. These 30 months do not include continuing training in and with their units or specialized training courses such as intelligence school, airborne school, or the Ranger course.

The first formal leadership training is the Basic Officer Leaders Course (previously known as Officer Basic Course), a 19-week course that teaches young officers small unit tactics and how to effectively lead a platoon in combat. Around year 5 of service, the next formal training step is the Captain's Career Course (CCC). The Captain's Career Course is a 22-week course that prepares officers to lead a company-sized element and effectively serve as a staff officer at the battalion level. At the 10-year mark, officers must complete the 10-month Intermediate Level Education course, formerly known as the Command & General Staff College (CGSC) in Ft. Leavenworth, Kansas, an accredited graduate-level program where students receive a Master of Military Arts and Sciences degree upon completion. The Intermediate Level Education course prepares rising officers to lead battalion-sized elements and task forces and serve as staff officers at the brigade and division level. It is at this level when infantry officers take a deep dive into the operational art of warfare and are expected to master battalion- and brigade-level tactics. The capstone of U.S. military education is the U.S. Army War College (known as Senior Service College/ SSC), taken by lieutenant colonels or colonels between year 16–25 of service. Over 10 months, select lieutenant colonels and colonels study strategic level content including unified operations, theater-level campaign planning, and national military and security strategy (4).

In a typical 25-year career (300 months), a U.S. Army officer receives thus a minimum of 30 months of formal military education to prepare themselves for effective and successful assignments at higher levels of command. This does not include all the additional training within the officer's specialty that can add several months to the total. For example, a tank officer will undergo the same formal training outlined above plus specialized training how to conduct combat operations as commander of a tank company, armored battalion, or armored brigade combat team. Thus, the U.S. military invests and reserves at least 10% (!) of the total time spent in a military career to formally train its leaders.

## Leadership levels in academic medical centers

Compared to the military, academic medical centers (AMC) and healthcare systems have far fewer organizational levels or echelons (Table 2 contrasts military and AMC echelons). The first leadership opportunities for physicians arise as leads of small teams or programs that often match the core clinical expertise of the faculty member. The leadership challenges for physician team leaders are not dissimilar to that of an infantry platoon leader and often focus on immediate people management, clinical operations, quality and safety and to some degree education and teaching.

The next, and first formal, organizational unit in an academic medical center is typically that of a section or division. Section heads or division chiefs are mostly senior faculty (associate professor or higher) and often have more than a decade of experience in the specialty. At the level of section or division, new leadership challenges arise: management of a large and often more diverse group of physicians and other healthcare professionals, oversight of and responsibility for the section/division budget and tripartite mission (clinical, education, and research), recruitment, human resources, and to some degree, faculty development. Unlike the military, physician leaders can and often chose to remain at the level of section/division chief and not pursue higher leadership roles, such as department chair or dean, so that they can continue to work predominantly within their clinical subspecialty.

At the department level, chairs are responsible for planning and implementing departmental strategy; fiduciary oversight of departmental finances; the complete spectrum of the tripartite mission, including clinical operations, undergraduate, graduate and post-graduate medical education, and research; human resources including management of diverse groups of employees (faculty, staff, trainees, healthcare professionals, researchers, etc.), to name a few core responsibilities. Chairs are expected to understand and align their departmental strategy with the overall strategy of the medical center and the university. They thus function at the interface between the operational and strategic level and have to navigate the often conflicting priorities from the hospital and university. Clinical departments often have a formal “headquarters” unit that may include an executive committee, business administration and support staff—in this regard the leadership structure of a clinical department is somewhat similar to that of an infantry battalion or brigade.

Leadership of an academic medical center—as dean, CEO, or in similar role—has been described as leading the most complex business organization mankind has invented. AMCs integrate the full spectrum of healthcare business operations with managing a medical school, often the largest academic unit in a university. Historically, academic medical centers operated a single or only a small number of interconnected hospitals on a single campus. However, over the last 20–30 years, AMCs have evolved into large healthcare systems that may comprise of a large number of diverse healthcare facilities over a wide geographic range, sometimes in multiple states and even international (e.g., Cleveland Clinic, UPMC). Thus, the leadership challenges of large AMCs are not dissimilar from leading a large Army unit such as a division or corps (Table 2).

## Physician leadership training

Academic physicians are not required to obtain formal leadership training before assuming a leadership role. This is true for team leaders all the way to deans. Nevertheless, many aspiring leaders do so on a voluntary basis. Many academic medical centers have begun to offer leadership training for promising or recently appointed section/division chiefs and vice chairs. In my own experience, Barnes-Jewish Hospital/BJC and Washington University school of Medicine offered a one semester long course, Friday and Saturday once a month, that covered the basics of healthcare finance, supply chain logistics, leadership and management essentials (5). This course was targeted mostly for mid-level leaders, and was open to academic physicians, nurse leadership and hospital management. It was co-taught by faculty from the business school. The University of Chicago Medical Center offers a somewhat similar program that is restricted to physician leaders (6). The Harvard T.H. Chan School of Public Health offers a 2-week immersive program for new clinical chairs (7). Other institutions, the Association of American Medical Colleges (AAMC), and many subspecialty organizations, have established similar programs and have recognized the need for focused physician leadership training and education (8–12). It is beyond the scope of this perspective to discuss the details of each program.

The challenges of many of these programs are two-fold: first, the participants have often markedly different management and leadership experience, and wildly different foundations in content knowledge (from total novice to highly experienced). This is unavoidable when one single course is intended cover the whole range of health care leadership, from section/division to dean-level management. Second, these bi-weekly/monthly semester-long courses often can only scratch the surface of the content. There is insufficient time to go deeply into any topic. This is especially a problem with healthcare finance and business operations, two domains in which most physician leaders have little knowledge or experience.

On the other hand, a substantial number of physicians enroll in a formal MBA or executive MBA program, which are rigorous but often require a serious 1-to-2-year time commitment. While MBA programs “go deep”, they are difficult to get the timing right. Too early in one's career and it's difficult to apply the knowledge in practice; too late in a career and it may be extremely hard to find the time to devote to an MBA program.

It would, thus, make more sense to establish a ladder-like professional academic leadership education program that starts at the team lead and ends at the dean or health-system level. A concept is provided in Table 3. The first program is for team leaders, for example a physician leading a heart failure program, a critical care unit, or a robotic surgery program. The goal of this program is how to become an effective team leader and the curriculum may include team management; clinical operations; achieving excellence in clinical care; QI/QA; fundamentals of healthcare finance. The next level would be a program for section/division chiefs and the curriculum may include managing people; fundamentals of HR; fundamentals of faculty affairs and development; section/division finance and business operations; graduate medical education; clinical operations; research management. The third level is for department chairs or vice chairs and the curriculum may include HR; faculty development and affairs; department finances and business operations; budgeting; controlling; contracting; department operations; strategy and implementation; GME; clinical operations; fundamentals of fundraising and philanthropy; QI/QA; healthcare law and regulations (local, state, federal); research management. The goal of this program is to serve effectively at the department level.

**Table 3:** Concept for professional physician leadership education.

	Leader	Duration of Course	Core Curriculum	Goals
Team	Team Lead	7–10 days	Management of small teams; clinical operations; achieving excellence in clinical care; QI/QA; fundamentals of healthcare finance;	Becoming an effective team leader
Section/Division	Chief/Head	2–3 weeks	People management; fundamentals of HR; fundamentals of faculty affairs and development; section/division finance; GME; clinical operations; research management;	Enabling the participant to effectively serve as section chief
Department	Chair	4 weeks	HR; faculty development and affairs; dept finances; budgeting; controlling; contracting; dept operations; dept strategy and implementation; GME; clinical operations; fundamentals of fundraising and philanthropy; QI/QA; healthcare law and regulations (local, state, federal); research management	Enabling the participant to effectively serve as department chair or assoc/vice chair.
Academic Medical Center/School of Medicine/ Hospital	Dean/CEO	4 weeks	Management of complex organizations; people management; strategy; operations; healthcare law and regulations (local, state, federal); AMC-level healthcare finance; UME, GME, CME; research management; higher education; marketing;	Enabling the participant to effectively serve as Dean, hospital CEO or senior officer in the Dean's office/hospital C-suite
Healthcare System	CEO	4 weeks	Same as AMC but focus on system-level	Enabling the participant to effectively serve as system CEO or in the system C-suite

The fourth level is for Deans and leaders at the AMC-level and the curriculum may include management of complex organizations; people management; strategy; operations; healthcare law and regulations (local, state, federal); AMC-level healthcare finance; UME, GME, CME; research management; higher education; marketing; philanthropy. For large healthcare systems, a fifth level may be necessary that will provide education how to effectively manage large healthcare systems (Table 3).

One could envision such program being offered through national organizations, such as AAMC, AMA or others. Academic medical centers would send their future leaders to these courses prior to taking leadership roles, which would establish a common standard of knowledge among leaders from different disciplines, backgrounds, and institutions. Each institution may then elect to supplement the general education with institution-specific education that can now focus exclusively on the nuances of the individual AMC while the general basics have already been covered. Such approach would tie in with established leadership competency models for healthcare managers, such as the National Center for Healthcare Leadership (NCHL) model (13, 14), and Healthcare Leadership Alliance (HLA) model (15).

Using the stepwise approach, the US military takes to train its leaders over a career—from small to large, from rather simple to highly complex—we may be able to offer academic physicians a strong foundation for success as leaders in academic medicine.
